# Post-procedure micro-CT analyses of coronary artery stenting in left main vessels of reanimated and perfusion-fixed human hearts

**DOI:** 10.1186/s12938-023-01090-2

**Published:** 2023-03-18

**Authors:** Thomas F. Valenzuela, Paul A. Iaizzo

**Affiliations:** Department of Surgery, University of Minnesota, Visible Heart®Laboratories, 420 Delaware St. SE, B172 Mayo, MMC 195, Minneapolis, MN 55455 USA

**Keywords:** Optical coherence tomography, Percutaneous coronary intervention, Drug-eluting stent, Coronary artery disease

## Abstract

**Background:**

Percutaneous coronary interventions (PCIs) within left main coronary arteries are high-risk procedures that require optimization of interactions between stent(s) and diseased vessels. Optical Coherence Tomography (OCT) is a widely accepted tool that enhances physicians’ ability to assess proper stent appositions during clinical procedures. The primary aim of this study was to develop complementary post-procedure imaging methodologies to better assess and interpret outcomes of left main PCI procedures, utilizing both reanimated and perfusion-fixed human hearts.

**Methods:**

PCIs were performed while obtaining OCT scans within the left main anatomies of six human hearts. Subsequently, each heart was scanned with a micro-CT scanner with optimized parameters to achieve resolutions up to 20 µm. Scans were reconstructed and imported into a DICOM segmentation software to generate computational models of implanted stents and associated coronary vessels. 2D images from OCT that were obtained during PCIs were compared to the 3D models generated from micro-CT reconstructions. In addition, the 3D models were utilized to create virtual reality scenes and enlarged 3D prints for development of “mixed reality” tools relative to bifurcation stenting within human left main coronary arteries.

**Results:**

We developed reproducible methodologies for post-implant analyses of coronary artery stenting procedures. In addition, we generated high-resolution 3D computational models, with ~ 20-micron resolutions, of PCIs performed within reanimated and perfusion-fixed heart specimens.

**Conclusions:**

Generated computational models of left main PCIs performed in isolated human hearts can be used to obtain detailed measurements that provide further clinical insights on procedural outcomes. The 3D models from these procedures are useful for generating virtual reality scenes and 3D prints for physician training and education.

## Background

Coronary artery disease is defined as the narrowing of coronary vessels caused by buildup of sclerotic fatty deposits, otherwise known as plaque. In cases of left dominant coronary circulation, it has been estimated that the left main (LM) coronary artery can supply > 75% of the left ventricular myocardium [1]. It has also been reported that patients presenting with LM stenoses ranging between 50% and 70% had projected 3-year survival rates of ~ 65%, whereas patients presenting with stenoses > 70% only had 3-year survival rates of ~ 40% [2]. Thus, in patients with significant LM disease, percutaneous coronary interventions (PCIs) with drug-eluting stents have been increasingly recognized as valid clinical revascularization procedures [3]. Because of such clinical severity, proper deployment of stents in the LM and their resulting strut appositions play a critical role in patient outcomes.

Recently, intra-procedural coronary imaging technologies have been developed and dramatically improved to allow interventional cardiologists the ability to view relative plaque depositions, lumen dissections, and/or stent appositions during various clinical PCI procedures. One tool that is gaining acceptance is the application of Optical Coherence Tomography (OCT) for such interventions. OCT uses a rotating glass fiber-optic system that tracks over a 0.014 guidewire until it is positioned in the desired location within the patient’s coronary anatomy. Once placed, a contrast flush is introduced into the OCT catheter, removing blood from the artery to allow for appropriate optical scanning; coherent infrared light is then directed and reflected within the tissue to create detailed images [4]. While OCT, in combination with fluoroscopy, continues to be a valuable tool in left main PCI cases, it is not universally available and is often reserved for the most complex cases.

A bifurcation PCI procedure and its long-term outcomes are heavily determined by multiple factors including, but not limited to: preprocedural planning, stent selection, available intraprocedural imaging (e.g., OCT or intravascular ultrasound), and physician experience. Experts in the field often identify *overall performance of the operator* as the key factor in successful bifurcation procedures [5]. Outcomes can be influenced by the clinician’s physical ability to perform the requisite technical steps, as well as experience and knowledge to identify potential complications to avoid pitfalls during a procedure. Therefore, it is imperative that cardiac interventionalists, both experienced and novice, are continuously trained and educated on the latest developments in bifurcation stenting strategies and intraprocedural tools. Fortunately, there is a growing selection of training modalities, ranging from silicone benchtop to simulator training models, which have been developed over the years to train PCI operators [5]. The advantageous and disadvantageous of the various training models currently available are well-summarized in the 16^th^ Consensus of the European Bifurcation Club. However, no model is perfect and thus continuous development of these existing tools is warranted.

For over 25 years, the Visible Heart^®^ Laboratories have been dedicated to translational research and advancements in cardiac research, devices, and education [6, 7]. We routinely perform PCI procedures, including many bifurcations, within reanimated large mammalian hearts [8]. On rare occasions, we have the privilege to conduct PCIs within reanimated human hearts [9]. Our laboratories provide a unique research platform, where PCIs can be performed using experimental devices, novel or approved procedural techniques, and/or multimodal imaging modalities, all with no risks to living patients. The primary aim of this study was to further develop complementary post-procedure imaging methodologies to improve access to users, and to better assess and interpret the outcomes of left main PCI procedures in clinically relevant disease states by utilizing both reanimated and perfusion-fixed human hearts.

## Results

**Fig. 1:**
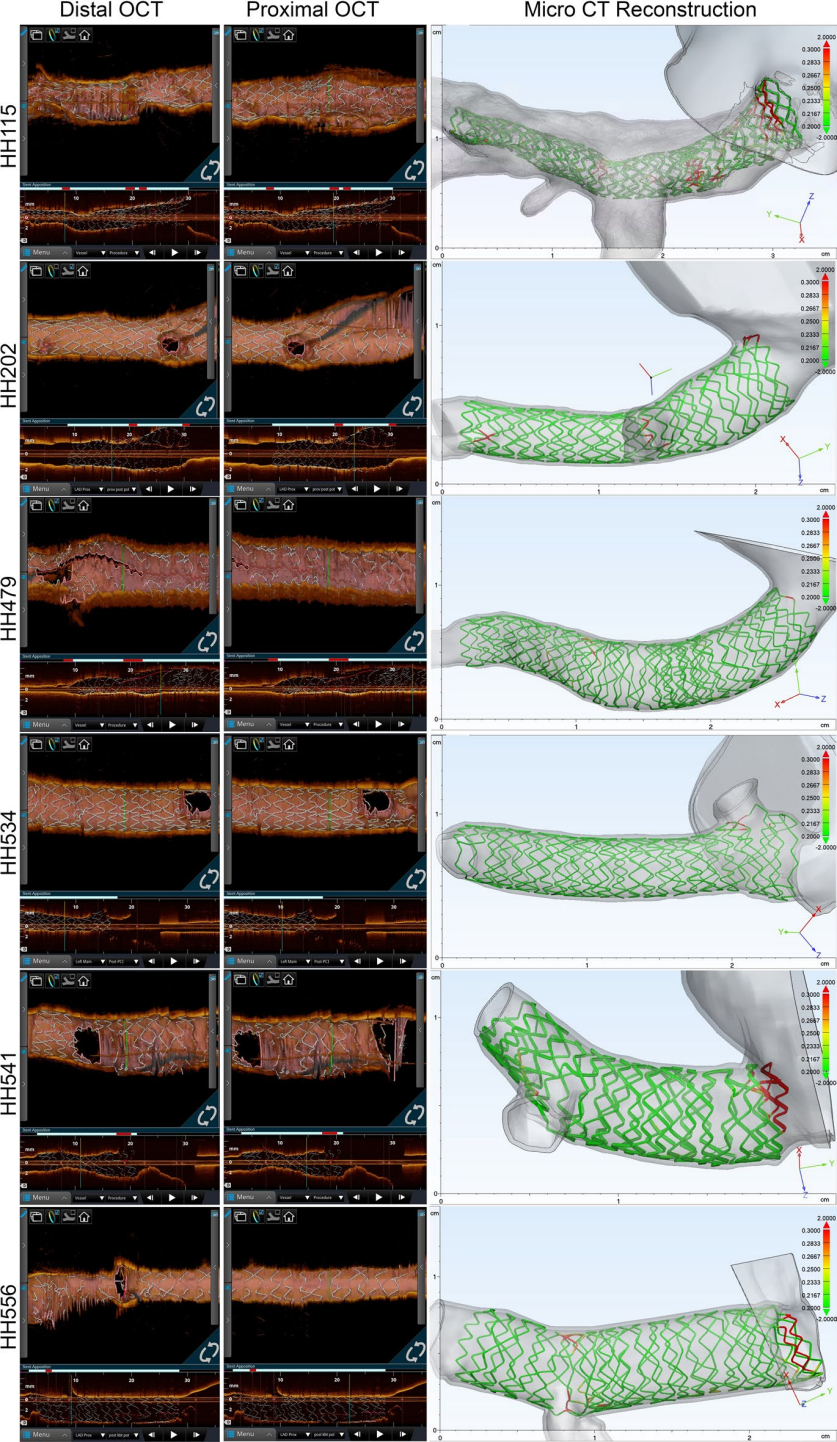
(Left) Series of 2D images showing distal and proximal portions of the stent obtained via Optical Coherence Tomography (OCT) imaging, using the Bifurcation Display, Stent Renderer, and Apposition Indicator function. (Right) Snapshots of the micro-CT 3D-generated computational model showing relative apposition analyses, to visually compare each stenting procedural outcome with OCT software

We successfully performed PCI procedures in six human LM coronary arteries employing Visible Heart^®^ methodologies, in both reanimated (*n* = 3) and perfusion-fixed (*n* = 3) hearts. These experimental approaches were deemed feasible and valuable for conducting preclinical research. We acquired OCT images immediately after each stenting procedure, then micro-CT was employed to scan all six hearts that were formalin fixed (i.e., 3 reanimated hearts were subsequently perfusion-fixed as well), using the same scanning parameters. Subsequently, the 2D OCT images were compared side by side with the 3D micro-CT reconstructions, and each micro-CT reconstruction accurately resembled its OCT counterpart, as shown in Fig. 1.

**Fig. 2:**
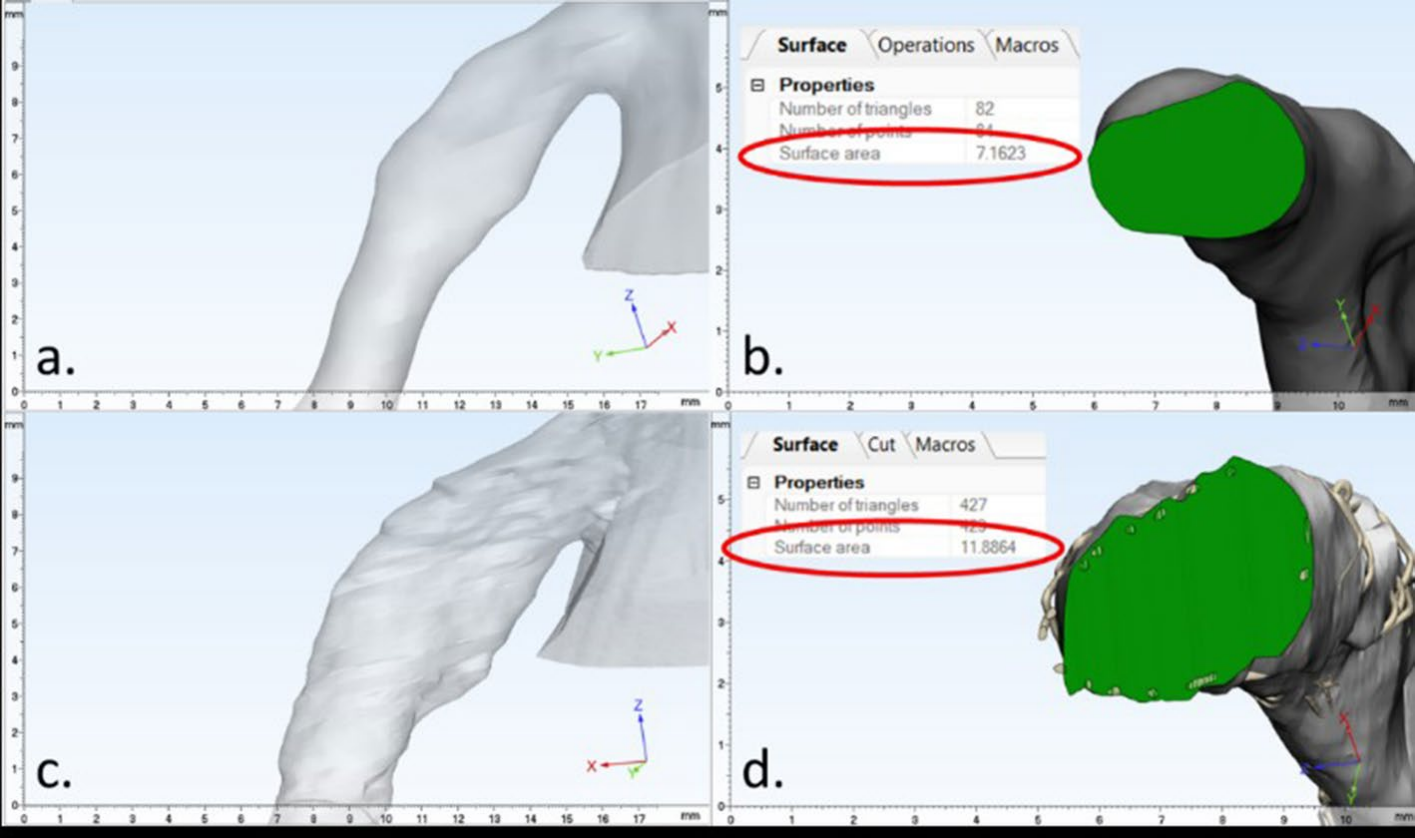
Using 3-Matic software, 3D renderings from micro-CT scans of vessels before and after PCI were used to determine changes in vessel dimensions post intervention. More specifically, cross-sectional areas were obtained 5 mm into the left main (LM) from the defined border of each coronary ostia of each heart specimen. An exterior view and cross section, with corresponding cross-sectional area, was taken for this location before (**a**, **b**) and after (**c**, **d**) PCI

The three reanimated specimens were relatively healthy and presented with no significant calcifications, as observed through endoscopic visualizations, OCT imaging, or micro-CT-generated computational models. However, varying degrees of vascular calcifications were found in the three perfusion-fixed specimens, as we intentionally selected these hearts with prior known histories of coronary artery disease. This was most notable in HH115 which presented with large amounts of plaque buildup. In this specimen, it was not possible to fully expand the deployed stent into a desired cylindrical shape via multiple balloon expansions employing > 20 atmosphere of balloon pressures. Despite the presence of vascular disease and previous perfusion fixation in formalin, these hearts still elicited significant increases, > 60%, in LM cross-sectional areas after stent implantations (Table 1). These values were calculated by measuring the cross-sectional areas of the given vessel lumens at 5 mm distances from the determined coronary ostia border that were determined before and after PCI (Fig. 2).

**Table 1:** Micro-CT measurements of left main lumen using blood volume pre- and post-percutaneous coronary intervention (computational assessments using 3-Matic)

	HH115	HH202	HH479
Pre-PCI area	7.16 mm^2^	10.54 mm^2^	12.25 mm^2^
Post-PCI area	11.89 mm^2^	17.14 mm^2^	19.80 mm^2^
% Increase	66%	63%	62%

Each heart exhibited varying areas prior to percutaneous coronary intervention (PCI) due to variability in heart size as well as extent of vessel disease, thus measurements were normalized by calculating overall percent increases in vessel lumen areas

In this study, we also sought to assess the relative effects of the formalin fixation process on a heart’s vessel geometries. This was achieved by taking OCT measurements post-PCI, while the heart specimen was < 12 h post-recovery (viable—fresh), to examine the LM lumen areas 2 mm distal to the deployed stents. We obtained additional OCT scans of the same specimens after formalin perfusion–fixation as means to evaluate lumen diameter changes induced by the fixation process. In addition, the same lumen areas post-fixation were measured via micro-CT to enable comparisons between measuring modalities. Reviewing the lumen areas in Table 2, we observed larger tissue desecration after formalin fixation in the healthy heart specimens, compared to those with previously noted heart disease. In addition, within the fixed specimens, some discrepancies in measurements between OCT and micro-CT were observed (Table 2), perhaps caused by methodological differences in how measurements were obtained. Ongoing investigations are currently underway in our laboratory to further assess the relationships between micro-CT and OCT measurements of various PCI procedures.

**Table 2:** Comparison of lumen measurements by Optical Coherence Tomography (OCT)

		2 mm distal OCT (mm^2^)
HH541 (healthy fresh)	Fresh post-PCI	7.32
	Formalin-fixed post-PCI	6.67
HH556 (diseased fresh)	Fresh post-PCI	9.55
	Formalin-fixed post-PCI	9.47

OCT measurements of the lumen at a distance 2 mm distal to the implanted stents. Comparisons were made in specimens with and without disease (reanimated heart that was subsequently formalin fixed) to evaluate lumenal changes associated with fixation process

**Fig. 3:**
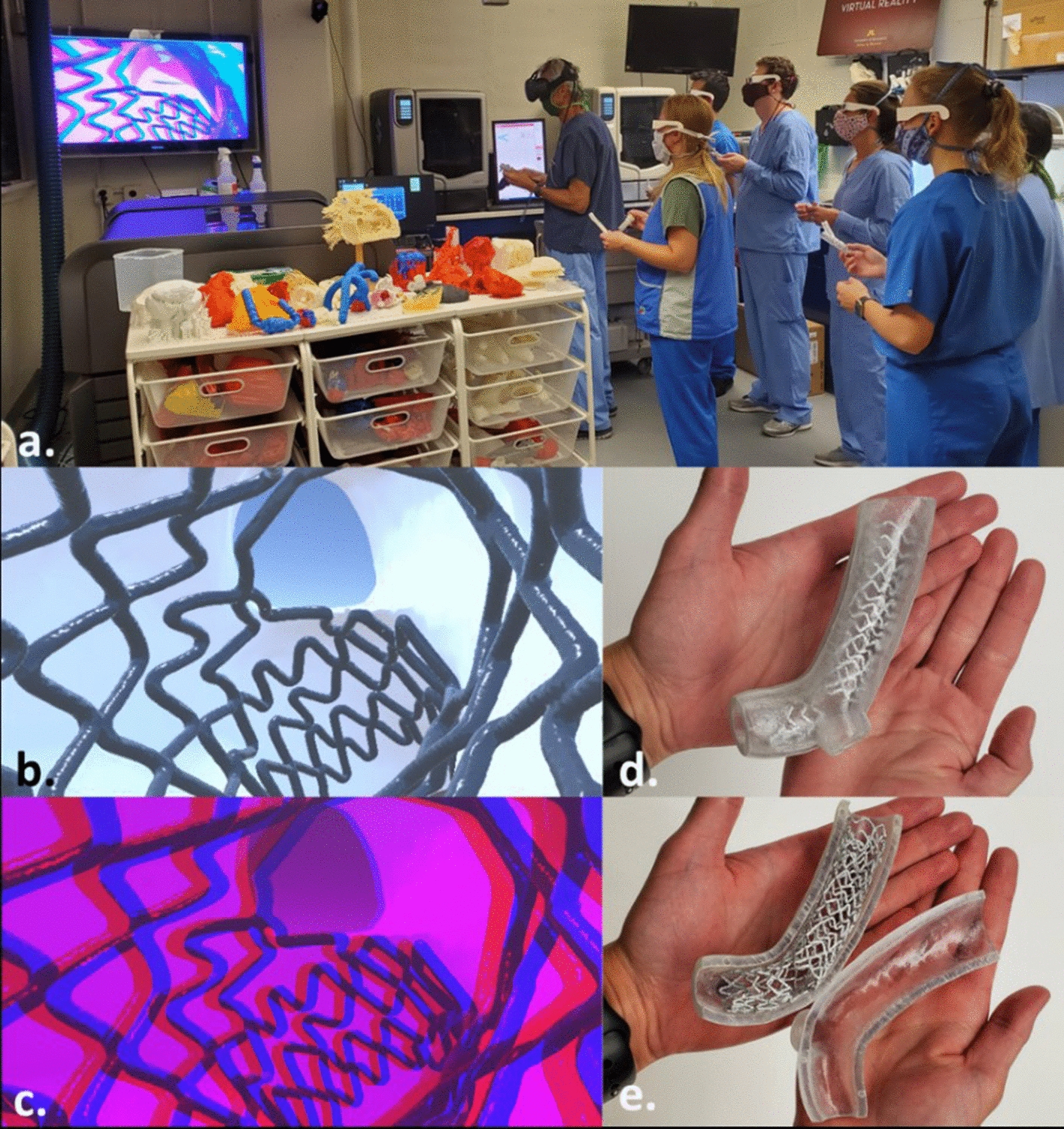
Virtual reality and 3D printing have been used as effective educational tools to enhance knowledge of bifurcation stenting interventions. In one application, the instructor can “fly around” the anatomy of a bifurcation stent (**a**, **b**), while trainees follow along within anaglyph visualization scenes (**c**). Simultaneously, mentors and trainees can hold 3D prints of the same model as viewed in the visualizations (**d**, **e**)

In addition, we used generated computational 3D models from micro-CT data sets to create “fly through” animations and develop virtual reality scenes (Fig. 3b). These scenes can enhance critical analyses of procedural outcomes and educate clinicians on methodological steps for optimizing bifurcation stenting. While feedback gathered to date from hundreds of novice and more experienced interventionalists has been positive, a formal investigation has not yet been conducted to evaluate the educational impact of these scenes. Notably, medical students have concluded that virtual reality augmented training experiences are beneficial for clinical preparation [10]. Uniquely, our laboratory has developed an approach to transform virtual reality scenes into anaglyph visualizations (Fig. 3c) in real-time, allowing multiple individuals to view PCI methodological scenes simultaneously (Fig. 3a). Free downloads of such scenes can be found in the Atlas of Human Cardiac Anatomy (http://www.vhlab.umn.edu/atlas/device-tutorial/stents/index.shtml). In addition, these models can be readily adapted for 3D printing as a unique tool that allows individuals to physically hold the same stent that they are flying through in virtual reality (Fig. 3d, e)—a “mixed reality” learning experience.

## Discussion

### Study limitations

For over 25 years, the Visible Heart^®^ Laboratories have routinely used reanimated swine hearts for a large variety of cardiovascular technological studies. We previously utilized implanted animal perfusion-fixed heart specimens to define the initial micro-CT scanning parameters used in this study of human hearts [11]. Through our initial attempts of scanning coronary stents in perfused tissue, it became apparent that we were not using enough voltage/power to penetrate through the struts, allowing it to be picked up by the scanner’s detector and causing a “halo-ing” effect. In the present study, by increasing the power employed for scanning, we reduced halo artifacts yet were initially unable to penetrate appropriately through the struts, thus resulting in hollowed cores. These streaking and shadowing artifacts of the struts yielded less than desirable 3D models. We further altered parameters and, once the optimized voltage and current values were determined, were able to finalize additional parameters such as number of projections and scanning durations to further reduce imaging artifacts; this allowed us to accurately depict the stent struts with a resolution of 20 µm. Nevertheless, we continue to improve these methodologies as well as those for perfusion fixation and orientating hearts in the scanner so we can detect different cardiac anatomies with similar densities (i.e., cardiac valves, endocardium, myocardium). We recently obtained and installed a micro-CT X3000 North Star Imaging scanner system (Rogers, MN, USA) in our laboratory to more easily perform such studies on all types of specimens.

We consider that the unique uses of formalin-fixed human hearts allows for improved imaging capabilities when performing complex PCI in more representative disease states, when compared to previous studies using animal trials [11, 12], silicone models [13], computational models [14], and/or simulators [15]. However, our preclinical approaches are not without limitations, as noted above. The perfusion-fixed human hearts were nonfunctioning, thus we were unable to recreate a sinus rhythm or vessel responses such as spasms or other anatomic variations throughout the cardiac cycle. However, we observed that diseased vessels in the reanimated human hearts (like in the patient with coronary artery disease), lose their compliance, and the perfusion–fixation process elicited little to no effects on the relative mechanical properties of the tissue studied. Nevertheless, our laboratory is conducting ongoing studies to further determine the effects of formalin fixation on diseased aortic and coronary tissues, i.e., by performing uniaxial and biaxial testing. These preliminary finding on formalin fixation indicate that we can likely utilize the 600 + valuable heart specimens within our Human Heart Library to perform similar preclinical benchtop experiments in real human cardiac anatomies, both normal and/or diseased.

Due to potential limitations of using formalin-fixed hearts, we also included fresh reanimated human hearts in the present study. The three human hearts in this study functioned with native sinus rhythms, enabling the relative recreation of vessel responses before, during, and after device deployments with representative hemodynamics. However, two of the three reanimated specimens did not present with coronary artery disease and likely had somewhat more compliant vessels (allowing for some degree of stent recoil) when compared to diseased vessels. This is because 10.5% of all hearts donated to the Visible Heart^®^ Laboratories have documented coronary artery disease, while only 92 (15%) of all hearts have met the criteria for reanimation. When we have the unique opportunity to reanimate a human heart, we make every effort to maximize what can be learned from these studies. Thus, we consider that the complementary use of reanimated and perfusion-fixed human hearts, as described here, is an important approach for studying device–tissue interfaces and current or new technologies.

### Creation of educational stenting modules

We have utilized these methodologies to generate high-resolution 3D computational models that offer unique mixed reality educational opportunities. For example, we used these reconstructed models of bifurcation stenting procedures to create a variety of enlarged 3D prints designed to better understand differences in various bifurcation techniques and their interaction with either calcifications (when present) and/or other complex associated anatomies. Furthermore, developed mixed reality educational modules can couple 3D prints and virtual reality scenes [16] that allow individuals or multiple users to “fly through” the same scenes [17] while simultaneously performing physical inspections of the detailed models [18]. In other words, these mixed reality teaching modules can be used for a multitude of educational endeavors, such as anatomical analyses, identifying clinical plaque deposition trends, assessing coronary stent designs, and/or developing computational fluid simulations (see free-access website “Atlas of Human Cardiac Anatomy” to visualize and download models and procedural videos; http://www.vhlab.umn.edu/atlas/index.shtml). Based on our experience, we believe that it is extremely helpful to develop these educational modules/materials to better engage individuals and stimulate learning. Such approaches can provide unique insights related to how interventional procedures may be applied in various clinical scenarios, and are thus valuable to students, clinicians, and/or medical device designers.

### Extension of application

We continue to perform additional PCI studies to further validate the accuracy of our novel micro-CT imaging. This study focused primarily on achieving reproducible scans of implanted coronary stents; however, our investigations continue to evaluate the final stent apposition via these micro-CT methodologies. We understand that having near perfect apposition long before the end of a PCI procedures is vital, especially when addressing issues such as wiring across a lesion to validate procedures. Such studies are also ongoing within perfusion-fixed human specimens in our laboratory. For example, we have started to perform step-by-step bifurcation procedures with imaging employed for each procedural step.

Furthermore, the methodologies described in this study are not limited to coronary stenting procedures. Rather they can be utilized for procedural testing of other cardiac devices to study device–tissue interactions with extremely high (~ 20 micron) resolution. For example, we are conducting studies in which various post TAVR–PCI procedures are performed within either reanimated or perfusion-fixed hearts on the Visible Heart^®^ apparatus; all hearts are subsequently micro-CT scanned using the same parameters provided here [19].

## Conclusions

In conclusion, we developed reproducible methodologies for post-implant analyses of coronary artery stenting procedures performed within reanimated and perfusion-fixed human hearts. Subsequently, we generated high-resolution computational 3D models to further investigate various device–tissue interfaces. These novel approaches for preclinical cardiac device testing can be used to generate virtual reality scenes and 3D printed models for a variety of educational mixed reality training. Our preclinical methodologies and scanning parameters are not limited to studying coronary stenting technologies, but have and can be applied to all types of medical device technologies.

## Methods

### Specimen procurement and selection

The Visible Heart^®^ Laboratories received viable human hearts and heart–lung bloc specimens for research via LifeSource, a nonprofit organ procurement organization (Minneapolis, MN, USA). The donors (donor families) gave consent for these organs to be used for scientific research purposes via LifeSource. The hearts used in this study were deemed nonviable for transplant due to advanced patient age, cardiac downtimes, identified poor cardiac function, and/or other reasons. All specimens were received as fresh viable tissues, along with donors’ relative cardiac–pulmonary clinical histories. We dissected lungs (when present) from each heart specimen while fresh, and carefully cannulated them for immediate reanimation, if specific criteria were met. Otherwise, hearts were placed within a formalin fixation apparatus [20] for a 24-h fixation period. The perfusion–fixation apparatus preserved hearts in their end diastolic shape, importantly keeping the aortas and coronaries dilated. After being perfusion fixed (including hearts initially reanimated), each specimen was placed in its own container and stored for future studies.

We performed PCI procedures and subsequent micro-CT scanning utilizing six human heart specimens (see Table 3 for detailed patient information). Three of the specimens exhibited adequate cardiac function prior to donation and were reanimated and eventually perfusion fixed, as described earlier. The remaining three hearts were received from LifeSource 2–11 years prior to this study. We selected these specimens due to noted histories of coronary artery disease and prior preprocedural imaging indicating the LM coronaries were patent enough to perform PCIs.

**Table 3:** Detailed patient information from donation records received from Lifesource

Heart #	Age/Gender	Weight(kg)	Clinical conditions	Cardiac conditions	Date received	Date of additional Study	Procedure/location
HH115	62 M	68.2	Diabetes, HTN	CAD	April 2009	11/2019	Stenting—provisional/left main
HH202	68 M	90	Diabetes, HTN, HLD	CAD, CABG, Stent (bare metal)	May 2011	2/2020	Stenting—provisional/left main
HH479	74 M	93	Diabetes, HTN, HLD	Mild aortic stenosis, CAD, Angioplasty (2004)	February 2018	2/2020	Stenting—provisional/left main
HH534	54F	57.3	Asthma, HTN	Family history of CAD	June 2019	Reanimated June 2019	Stenting—provisional/left main
HH541	73F	85.6	HTN	N/A	August 2019	Reanimated August 2019	Stenting—provisional/left main
HH556	60F	61.5	HTN, CM	CAD	January 2020	Reanimated January 2020	Stenting—provisional/left main

Clinical and cardiac conditions were obtained from next-of-kin interviews, pre-recovery imaging, and additional imaging performed after hearts were received. *CABG* coronary artery bypass graft, *CAD* coronary artery disease, *CM* cardiomyopathy, *HLD* hypersensitivity lung disease, *HTN* hypertension

### Coronary intervention in isolated hearts

**Fig. 4:**
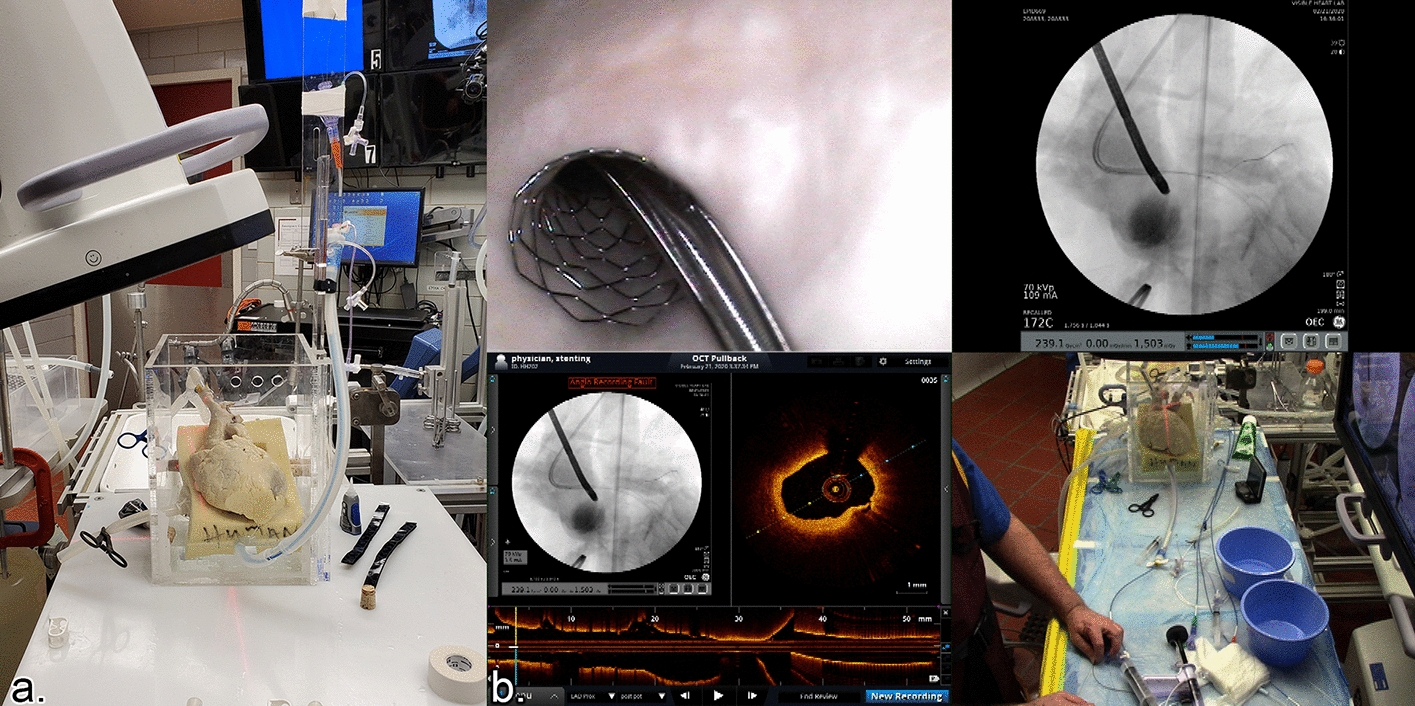
**a** Each perfusion-fixed isolated human heart was placed in a custom-made acrylic box for perfusion, utilizing fluoroscopy. **b** Quad-split of bifurcation showing endoscopic imaging, fluoroscopy, Optical Coherence Tomography images, and operator hand manipulations during percutaneous coronary intervention procedure

We performed PCI procedures in three reanimated hearts (HH534, HH541, HH556) using Visible Heart^®^ methodologies [21]; subsequently each heart was carefully removed from the apparatus and perfusion fixed so as not to damage the newly implanted stent(s). Previously fixed hearts (HH115, HH202, HH479) were rinsed for 24 h, re-cannulated, and placed in an acrylic box, where they were attached to a Langendorff static perfusion apparatus (Fig. 4a) that continuously perfused the aortas and coronaries with water. The advantages of utilizing Visible Heart^®^ methodologies, while performing PCIs were as follows: (1) endoscopic cameras enabled direct visualization of each procedural step; (2) there were no risks to any living patient, thus unlimited fluoroscopy could be used; and (3) OCT could be utilized as many times as desired as there was no need to expedite the procedures.

Each PCI procedure was guided and recorded simultaneously by 2.4 mm and 4 mm endoscopic cameras (Olympus, Tokyo, Japan), OEC Elite Fluoroscopy (GE, Boston, MA, USA), and episodic OCT imaging (OPTIS™ Mobil System, Abbot Vascular Inc., Abbott Park, IL, USA), as shown in Fig. 4b. The combination of these imaging modalities nearly simultaneously would not be possible without the use of a clear perfusion solution (Krebs–Henseleit buffer for reanimated hearts and water for fixed hearts) that continuously circulated through the apparatus. All PCIs were conducted using Resolute Onyx drug-eluting stents, compliant and non-compliant Euphora balloons, and Cougar XT guidewires (Medtronic, Santa Rosa, CA, USA).

### OCT imaging during PCI

After stent implantations, we captured OCT scans using OPTIS System and Dragonfly™ Imaging Catheters (Abbott Vascular). The automatic pullback system captured 540 frames over a scanning trajectory of 54 mm with a 5 mm penetration distance, to capture the highest resolutions possible (~ 100 µm). Since specimens were continuously perfused using clear solutions, no contrast injections were needed or administered during OCT scanning.

Following ex vivo stenting, the three reanimated hearts were perfusion fixed and then placed in formalin containers for long-term preservation. All OCT image data sets were post-processed to identify both distal and proximal portions of the implanted stents and then stored as 2D images. Since intracoronary OCT is a widely accepted method of imaging implanted stents, the images collected were later used to compare to the micro-CT reconstructions.

### Micro-CT scanning

**Fig. 5:**
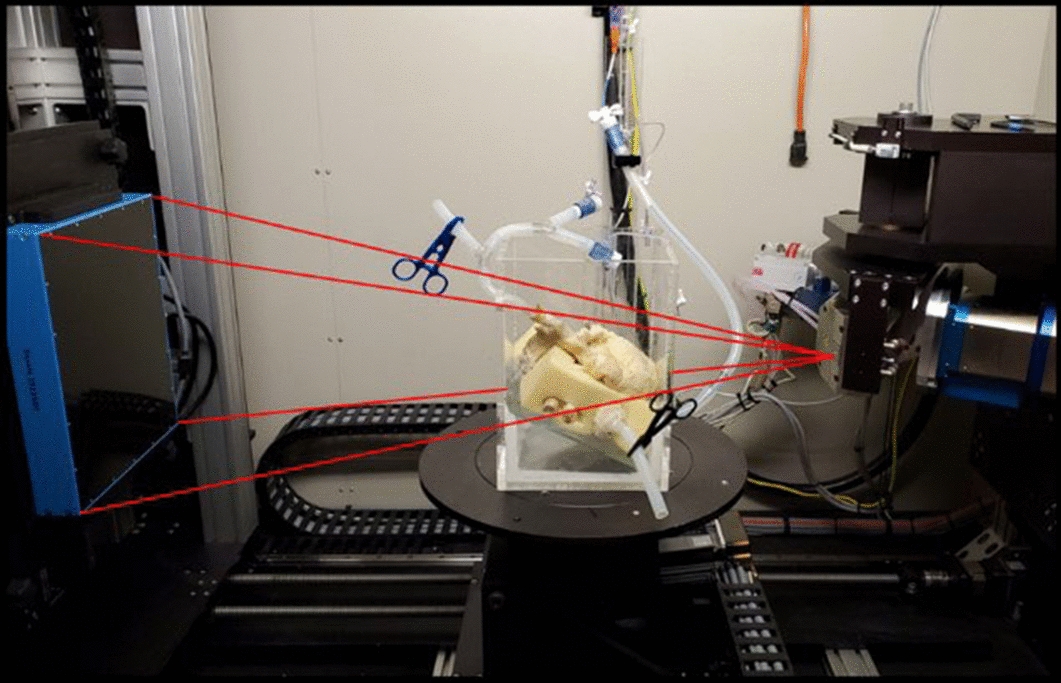
Same plexiglass case used for performing percutaneous coronary interventions was then used for micro-CT scanning. Tubes were disconnected from the perfusion apparatus and clamped, so fluid was not spilled during scanning

**Fig. 6:**
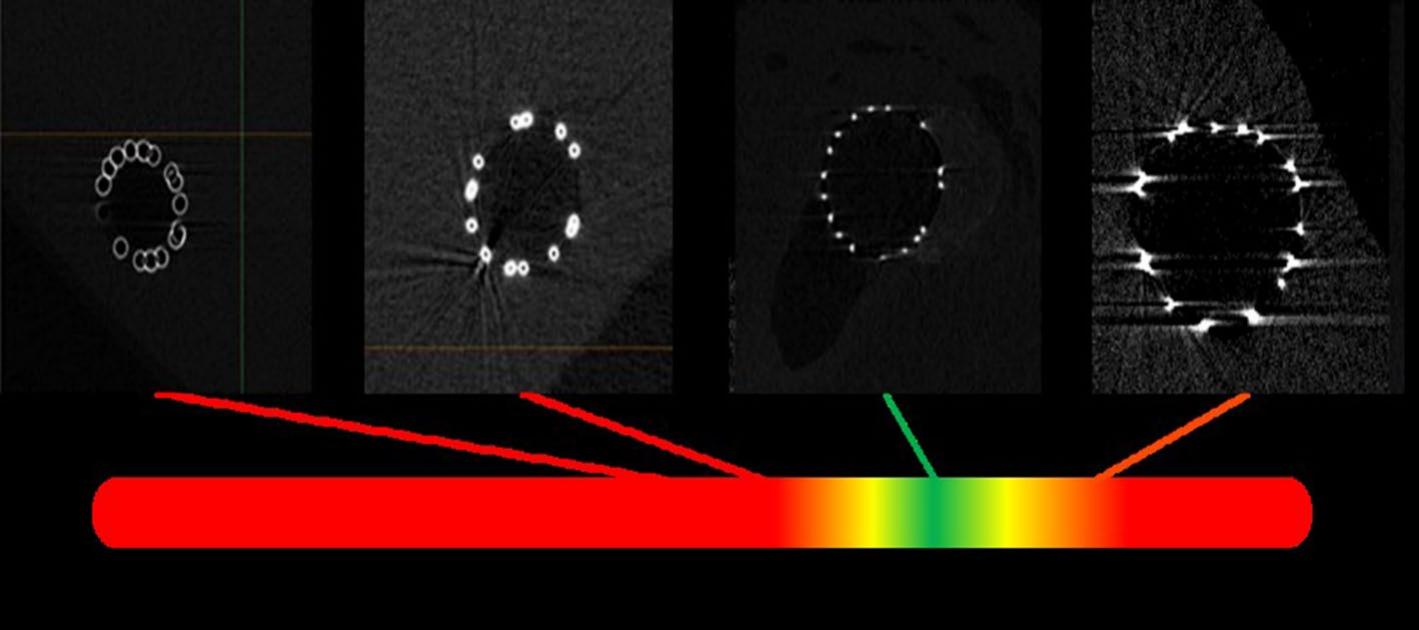
Initial attempts began with lower voltages and power that was unable to penetrate through the stent struts, resulting in outlining of the struts with black center cores. Too much voltage and power caused significant streaking in the stent struts, and further refinement of the parameters was needed to distinguish between the stent, tissue, lumen, and calcification

Before micro-CT scanning, the specimens were rinsed in water for a minimum of 24 h to remove traces of formalin before handling and/or transportation. Once thoroughly rinsed, each heart was placed within the specially constructed plexiglass container and then scanned using an X5000 micro-CT scanner (North Star Imaging). All heart specimens were carefully perfusion fixed to elicit an end diastolic shape for all four chambers (maximally filled) prior to scanning, because the internal space of the scanner did not readily allow for our perfusion system to be used during scanning. Each heart was placed in the scanner, as shown in Fig. 5, and imaging was performed utilizing the following parameters to achieve approximate isotropic voxel sizes of 20 × 20 × 20 µm: 170 kV tube voltage, 144 µA tube current, 24.5 isowatts, and 1500 radiograph images captured throughout a ~ 15-min scanning duration. We selected these parameters after numerous iterations and scanning trials, all utilizing Resolute Onyx stents implanted in swine heart coronaries, to optimize scanning resolutions while minimizing streaking, shadowing, and/or scanning artifacts. Figure 6 shows the progression of scanning parameters trialed, until we finalized the optimal parameters used in this study. Once scans were completed, each heart specimen was returned to the laboratory and placed in its respective formalin container. Imaging data sets were then reconstructed using North Star Imaging’s reconstruction software, into 8-bit 2D images (*.tiff*), for future analyses as described below.

### 3D reconstructions

We imported.tiff files from micro-CT scans into the DICOM analysis software Mimics (Materialise, Leven, Belgium), where they were computationally “stacked” to form 3D volumes from 2D images [22], followed by further post-processing. Using Mimics, for each heart’s image data set, we generated a high-density “mask” to segment out the higher density portions of the scan, i.e., the cobalt alloy shell and platinum iridium core of the Resolute Onyx. We manually created additional masks to segment out the vessel blood volumes and tissues. For subsequent analyses, each generated model consisted of a portion of the aortic wall, left coronary ostia, LM vessels, coronary stent(s), and proximal portions of left circumflex artery and left anterior descending coronaries. Once these models were generated, we used assessment tools in Mimics to measure the relative lumen areas. The 3D models were then exported from Mimics to be rendered as virtual reality scenes using a video game design software (Unity, Unity Technologies, San Francisco, CA, USA) that allowed for further visual inspections of LM stenting outcomes.

## Disclaimer

Approximately 5–7% of coronary angiography patients present with a left main lesion, with 60% of these patients eligible for PCI. The Resolute Onyx drug-eluting stent is currently CE marked for both left main and bifurcation stenting; however, to date, it is not approved in the United States and the FDA considers any left main stenting to be off-label.

## Data Availability

All data generated or analyzed during this study are included in this published article.
